# IL-23 Dependent and Independent Stages of Experimental Arthritis: No Clinical Effect of Therapeutic IL-23p19 Inhibition in Collagen-induced Arthritis

**DOI:** 10.1371/journal.pone.0057553

**Published:** 2013-02-28

**Authors:** Ferry Cornelissen, Patrick S. Asmawidjaja, Adriana M. C. Mus, Odilia Corneth, Kristine Kikly, Erik Lubberts

**Affiliations:** 1 Department of Rheumatology, Erasmus Medical Center Rotterdam, Rotterdam, The Netherlands; 2 Department of Immunology, Erasmus Medical Center Rotterdam, Rotterdam, The Netherlands; 3 Eli Lilly and Company, Indianapolis, Indiana, United States of America; Institut national de la santé et de la recherche médicale (INSERM), France

## Abstract

IL-23p19 deficient mice have revealed a critical role of IL-23 in the development of experimental autoimmune diseases, such as collagen-induced arthritis (CIA). Neutralizing IL-23 after onset of CIA in rats has been shown to reduce paw volume, but the effect on synovial inflammation and the immunological autoimmune response is not clear. In this study, we examined the role of IL-23 at different stages of CIA and during T cell memory mediated flare-up arthritis with focus on changes in B cell activity and Th1/Th17 modulation. Anti-IL-23p19 antibody (anti-IL23p19) treatment, starting 15 days after the type II collagen (CII)-immunization but before clinical signs of disease onset, significantly suppressed the severity of CIA. This was accompanied with significantly lower CII-specific IgG1 levels and lower IgG2a levels in the anti-IL-23p19 treated mice compared to the control group. Importantly, neutralizing IL-23 after the first signs of CIA did not ameliorate the disease. This was in contrast to arthritic mice that underwent an arthritis flare-up since a significantly lower disease score was observed in the IL-23p19 treated mice compared to the control group, accompanied by lower synovial IL-17A and IL-22 expression in the knee joints of these mice. These data show IL-23-dependent and IL-23-independent stages during autoimmune CIA. Furthermore, the memory T cell mediated flare-up arthritis is IL-23-mediated. These data suggest that specific neutralization of IL-23p19 after onset of autoimmune arthritis may not be beneficial as a therapeutic therapy for patients with rheumatoid arthritis (RA). However, T cell mediated arthritis relapses in patients with RA might be controlled by anti-IL-23p19 treatment.

## Introduction

IL-23 is a heterodimeric cytokine consisting of a p40 subunit, shared with IL-12, and a p19 subunit that is unique to IL-23 [1], [2]. Together with the IL-12Rβ1 receptor, the IL-23 receptor (IL-23R) chain forms a functional receptor for IL-23 [3] that is expressed on T cells, NK cells, monocytes and dendritic cells [1], [3]. However, IL-23R is not expressed on precursor T cells, suggesting that IL-23 signaling is not involved in the primary differentiation of naïve T cells [4]. The signaling of IL-12 and IL-23 leads to the activation of both overlapping and divergent signal transduction pathways and pathological roles in experimental arthritis [3]. IL-23 is elevated in many autoimmune diseases, such as psoriasis, rheumatoid arthritis (RA) and multiple sclerosis (MS) [5], [6]. IL-23 transgenic mice develop systemic inflammation, including inflammation of the skin and small and large intestine [7], highlighting the role of this pathway in promoting the activation of effector T cells and sustaining of inflammatory tissue responses.

The role of IL-23 in the development of autoimmune collagen-induced arthritis (CIA) has been shown using IL-23p19 knockout mice. These mice did not develop CIA compared to IL-23 sufficient controls [8]. In these IL-23p19 deficient mice, no IL-17 producing cells were detected while the proportion of IFN-γ producing cells was unaltered [8]. This indicates that IL-23 is involved in the generation of IL-17 producing T cells in vivo [2]. Furthermore, neutralizing IL-23 after onset of CIA in rats has been shown to reduce paw volume [9], but the effect on synovial inflammation and the immunological autoimmune response need to be elucidated.

Here, we investigated the role of IL-23 during different stages of autoimmune CIA by utilizing an IL-23p19 specific antibody. When anti-IL-23p19 was given after CIA onset, arthritis severity was not ameliorated. However, when anti-IL-23p19 was administrated after type II collagen (CII)-immunization but before clinical CIA onset, significantly less severe disease was observed. Finally, we show in a memory T cell dependent antigen-induced arthritis model that IL-23 is essential for the development of flare-up arthritis. In this model, synovial expression of IL-17A and IL-22 but not IFN-γ was markedly lower in the anti-IL-23p19 group compared to control, highlighting the role of IL-23 in memory T cell driven flare-up arthritis. Together, these data showed IL-23 dependent and independent stages during CIA and revealed that IL-23 is not a critical factor during the effectors stage of CIA. In contrast, memory T cell mediated flare-up arthritis is IL-23 dependent.

## Results

### IL-23 does not Enhance CII-specific IL-17A Production by CD4+ T cells

**Figure 1:**
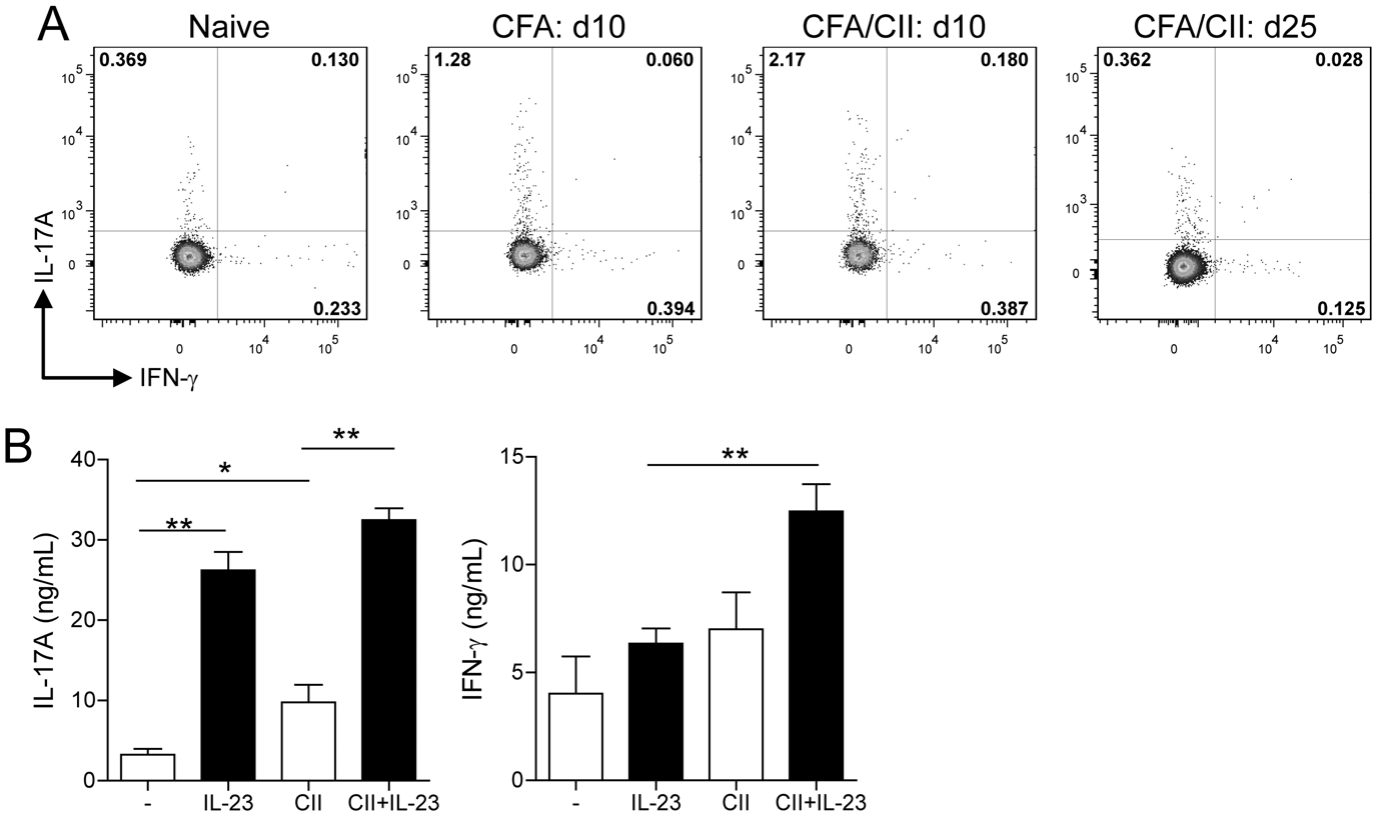
IL-23 does not enhance CII-specific IL-17A production. (**A**) DBA/1 mice were immunized with CFA only, with CII/CFA or left untreated. At days 10 (CFA and CFA/CII) and 25 (CFA/CII) post-immunization, splenocytes were isolated and assessed for intracellular expression of IL-17A and IFN-γ. Numbers in quadrant indicate percentage positive cells in that quadrant. Plots are representative of n = 3–6 per group. (**B**) IL-17A and IFN-γ secretion levels after antigen (CII) specific restimulation of purified splenic CD4+ T cells with irradiated APCs in the absence (−) or presence of exogenous IL-23. Data are the mean +SEM from n = 3 mice per group and *P<0.05; **P<0.01; ***P<0.001 as calculated by Mann-Whitney U test.

To profile the kinetics of Th1 and Th17 cells during collagen-induced arthritis (CIA), splenocytes were isolated from type II collagen (CII)-immunized DBA/1 mice at various time points post-immunization (p.i.) and assessed for intracellular cytokines by flow cytometry. At day 10 p.i. the highest proportions of total IL-17A+ and IL-17A+IFNγ- CD4+ T cells were observed as compared to naïve (non-immunized) mice as well as to mice 25 days p.i and CIA-diseased mice (Figure 1A). However, injection of CFA only also induced a clear population of IL-17+IFNγ- CD4+ T cells though lower than that observed in CII/CFA-immunized mice. This shows the generation of CD4+ IL-17A-expressing T cells during CIA.

Since IL-23p19 deficient mice revealed hardly any collagen (CII)-specific IL-17 producing CD4+ T cells [8], we were interested whether IL-23 is still required for CII-primed CD4+ T cells to produce IL-17A. Therefore, we evaluated the role of IL-23 on CII-specific cytokine production by stimulating purified CD4+ T cells taken from mice 10 days p.i. with CII/CFA (Figure 1B). CII-specific restimulation led to a significant increase of IL-17A secretion compared to non-restimulated CD4+ T cells. No IL-23 levels could be measured in these cultures and adding anti-IL-23p19 antibody had no effect on IL-17 production (data not shown). However, adding IL-23 clearly enhanced the production of IL-17A in both the non-restimulated and the CII-restimulated cultures but no significant difference was observed between both groups supplied with IL-23 showing that IL-23 does not contribute to the production of CII-specific IL-17A. Interestingly, CII-specific restimulation did not enhance the secretion of IFN-γ in CD4+ T cells (Figure 1B), however, an increased production of IFN-γ was noted when IL-23 was added in the CII restimulated CD4+ T cells compared to the un-restimulated cultures (Figure 1B). Importantly and in contrast to the dominant role of IL-23 in the development of collagen-specific CD4+IL-17+ T cells, these data suggest that IL-23 is not required for CII-primed CD4+ T cells to produce IL-17A in contrast to IFN-γ upon antigen-specific restimulation.

### Lower Severity of CIA after Neutralizing IL-23 during Arthritis Onset

**Figure 2:**
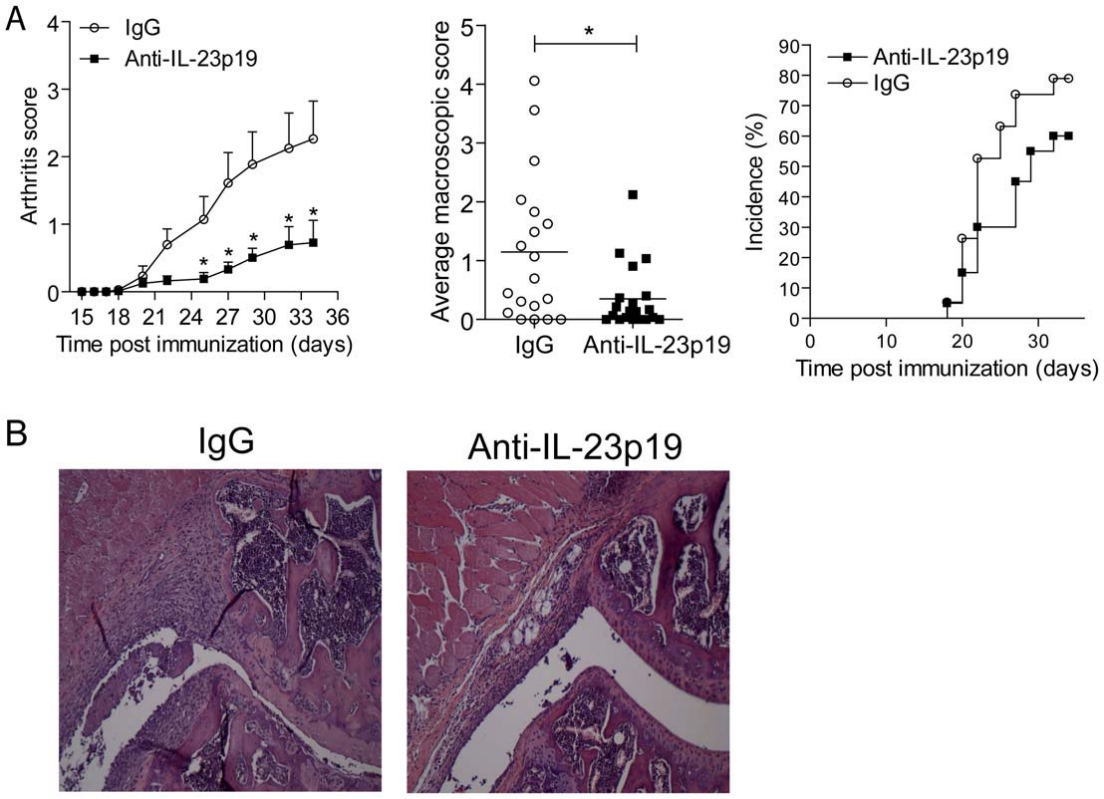
Administration of anti-IL-23p19 before onset prevents full-blown CIA. (**A**) DBA/1 mice were immunized with CII/CFA and three weeks later mice received a booster-injection. On days 15, 22 and 29 either anti-IL-23p19 (filled squares) or control antibody (open circles) was given intra-peritoneally. Macroscopic score (+SEM) and the average macroscopic score (average macroscopic score per individual mouse of all time points assessed, assessed by student t-test) of n = 20 mice per group from 2 independent experiments is shown, as well as the incidence.

To investigate the role of IL-23 during different stages of CIA, CII/CFA-immunized DBA/1 mice were randomly separated into two groups and treated with a murine anti-murine IL-23p19 antibody or isotype control antibody at days 15, 22 and 29 p.i. At day 21 p.i., a booster-injection was given according to our CIA protocol. Significantly less severe CIA developed in the anti-IL-23p19 treated group compared to control as assessed by the arthritis score and statistically tested at each individual time point over time by using the area under the curve (AUC), and histologically (Figure 2A,B). The disease incidence was not significantly different between the two groups and no difference in the day of onset was observed (Figure 2A).

### Reduced Levels of CII-specific Antibodies in Anti-IL-23p19 Treated Mice

**Figure 3:**
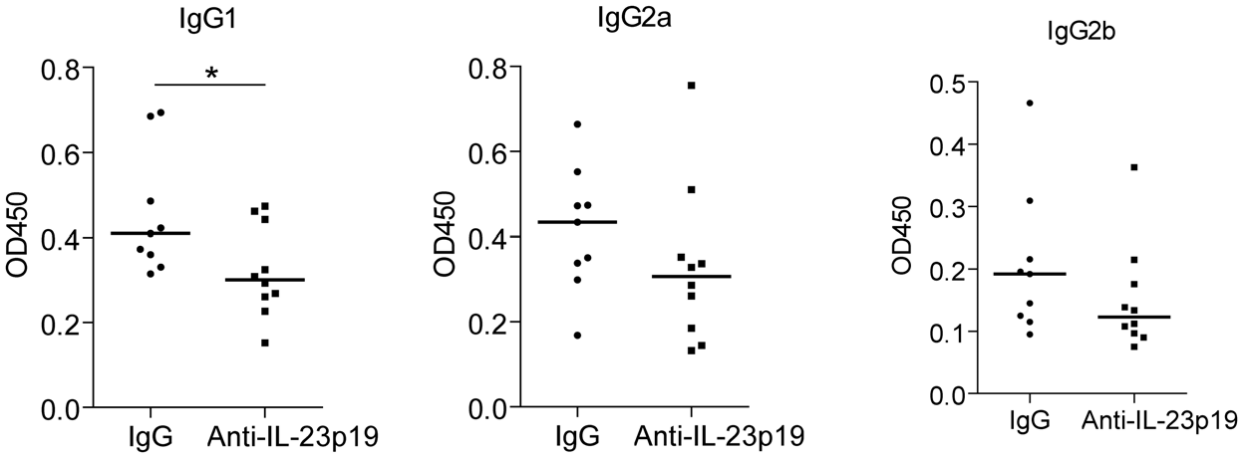
Decreased production of anti-CII antibodies in mice treated with anti-IL-23p19. At day 35, at the end of the experiment as shown in Figure 2A, serum was collected and anti-CII antibody levels of the IgG1, IgG2a and IgG2b subclasses were determined in sera. Data are shown for each individual mouse or as the mean +SEM of n = 10 mice per group and *P<0.05; **P<0.01; ***P<0.001 as calculated by Mann-Whitney U test.

To determine the effect of anti-IL-23p19 treatment on auto-antibody production, serum was collected at day 35 and anti-CII-specific IgG antibodies were measured. As shown in Figure 3, IgG1 levels were significantly lower in the anti-IL-23p19 treated group compared to controls. Additionally, IgG2a and IgG2b levels were lower; however, no statistically significant difference was reached.

### No Therapeutic Effect of Neutralizing IL-23 after Onset of CIA

**Figure 4:**
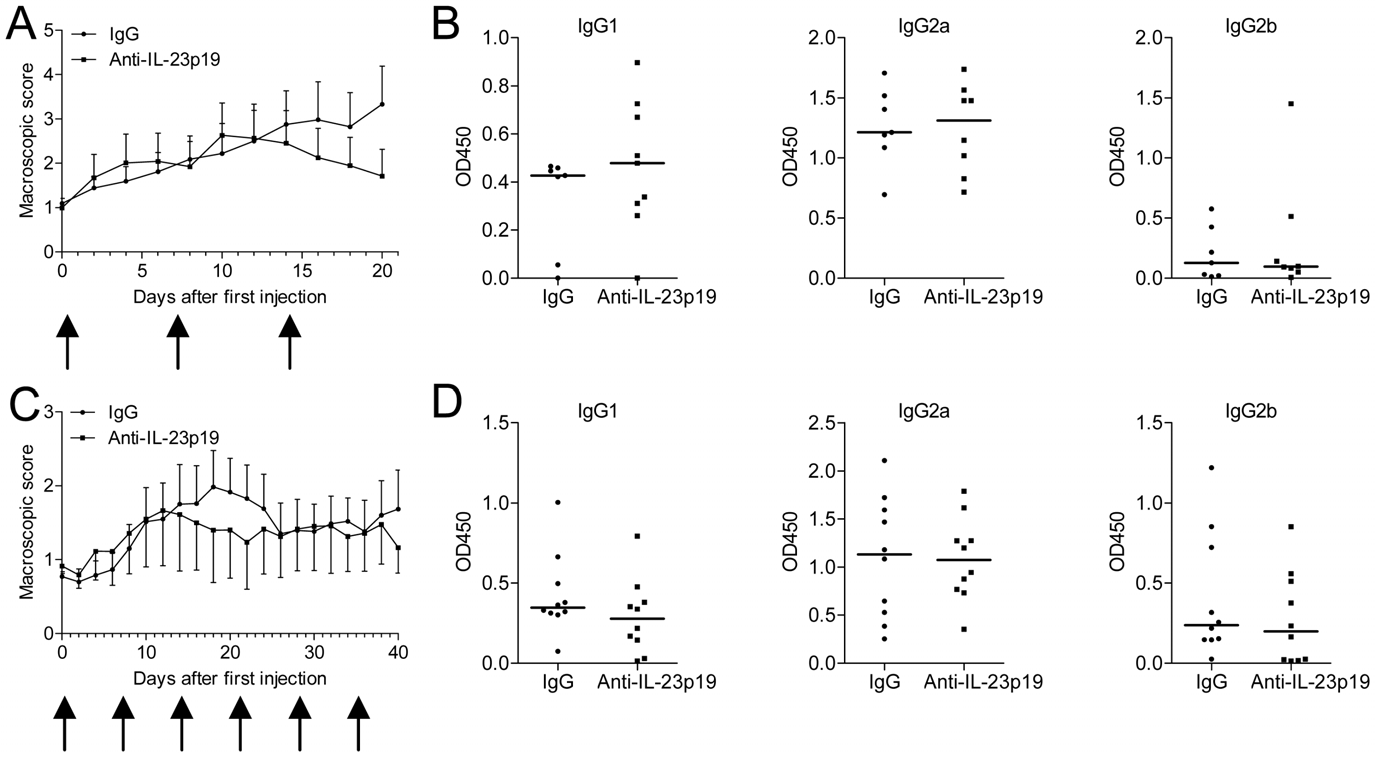
Neutralizing IL-23 after onset of CIA does not ameliorate disease activity. CIA was induced in DBA/1 mice and directly after the first signs of CIA anti-IL-23p19 or control antibody was injected when an arthritis score between 0.5–1.5 was observed. (**A** and **C**) Arthritis severity was scored macroscopically. Numbers on the x-axis indicate the day after the first injection with either antibody and the arrows indicate each time point an injection was given. Data are from (**A**) n = 8 and (**B**) n = 10 mice per group. (**B and D**) At the end of each experiment, serum was collected and anti-CII antibody production of the IgG1, IgG2a and IgG2b subclasses was measured.

Next, we asked whether anti-IL-23p19 has a therapeutic effect in CIA. To evaluate this, mice were treated with anti-IL-23p19 or control antibody weekly for 3 weeks after the first signs of CIA. Anti-IL-23p19 did not significantly suppress the arthritis score, compared with isotype control-treated mice (Figure 4A), and no differences in anti-CII specific IgG antibody levels were noted between the two groups (Figure 4B). To determine if longer treatment with anti-IL-23p19 would lead to a significant improvement in the arthritis score, anti-IL-23p19 or control antibody was administrated for a period of 6 weeks after disease onset. However, no ameliorative effect was observed (Figure 4C) after blockade of IL-23 compared to control and this also did not affect the production of anti-CII antibodies (Figure 4D). Together, these data indicate that specific neutralization of IL-23 after CIA onset has no beneficial effect on the CII-antibody titer or clinical score of CIA.

### IL-23 is Critically Involved in Memory T cell Mediated Arthritic Flare

**Figure 5:**
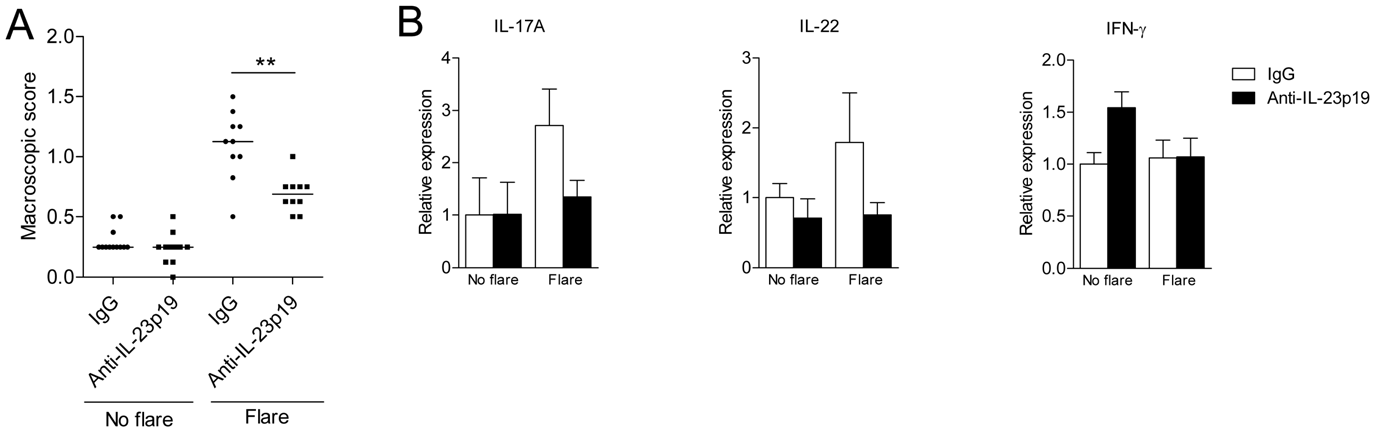
IL-23 plays a critical role in the arthritis flare-up reaction. In C57BL/6 mice, antigen-induced arthritis (AIA) was induced. Four weeks after the induction of AIA either IgG control or anti-IL-23p19 was administrated intra-peritoneally weekly for 5 weeks. One day after the final injection mice were given an intra-articular injection with mBSA to induce a flare-up or were left untreated. (**A**) Twenty-four hours later, knee joints were assessed macroscopically on a 0–2 scale. Each symbol represents data from an individual knee joint and the horizontal line depicts the median. Data are from n = 5 mice per group assessing both knee joints. *P<0.05; **P<0.01; ***P<0.001 by Mann-Whitney U test. (**B**) Cells from the knee-joints were isolated, RNA was extracted and gene expression for the indicated genes was quantified by quantitative RT-PCR and normalized for GAPDH. Data are the mean+SEM from 5 mice per group.

CIA pathology is initiated by CII-specific helper T and B cells which in turn lead to the generation of anti-CII-specific IgG producing plasma cells and the formation of pathogenic immune-complexes (ICs) [10]. To further dissect the role of IL-23 on reactivation of memory T cell mediated pathology, we utilized the mBSA-driven antigen-induced arthritis (AIA) flare-up model. Mice were immunized with mBSA/CFA and one week later an intra-articular injection with mBSA was given to induce a primary mono-arthritis which typically lasts for 3 weeks. After recovery from the primary arthritis, mice received five weekly injections with a murine anti-murine IL-23p19 antibody or isotype control antibody. One day after the last antibody injection, a local arthritic flare-up was induced by a second intra-articular injection with mBSA and the following day mice were sacrificed and the arthritis severity was assessed macroscopically. Mice treated with anti-IL-23p19 developed significantly lower arthritis scores compared with control (Figure 5A). To gain insight in the effect of IL-23 neutralization, RNA from knee-infiltrating cells was isolated and gene transcription was determined by quantitative RT-PCR. Synovial IL-17A and IL-22 expression was lower in anti-IL-23p19 treated mice compared to control, although not reaching statistical significance (Figure 5B). In contrast, synovial IFN-γ expression was similar between the two groups (Figure 5B). These data show that anti-IL-23p19 treatment significantly suppressed antigen-induced flare-up arthritis.

## Discussion

In the present study, we showed that anti-IL-23p19 antibody treatment, started after the onset of CIA, did not ameliorate arthritis severity. However, when anti-IL-23p19 antibody was administrated after type II collagen (CII)-immunization but before clinical signs of CIA onset, significantly less severe disease was observed. Finally, we showed in a memory T cell dependent antigen-induced arthritis model that IL-23 is essential for the development of flare-up arthritis. In this model, synovial expression of IL-17A and IL-22 but not IFN-γ was lower in the anti-IL-23p19 group compared to control, highlighting the role of IL-23 in memory T cell driven flare-up arthritis.

In contrast to our observation that neutralizing IL-23p19 after onset of CIA was not effective, Yago et al have claimed that anti-IL-23 antibody administration after onset of CIA in rats significantly reduced paw volume and attenuated CIA [9]. However, the data presented by these authors did not show a significant difference in arthritis score between anti-IL-23 treated rats and controls rats when they started treatment after onset of CIA [9]. Here, in the present study we clearly showed in repeated independent experiments no beneficial clinical effect of CIA when anti-IL-23 was administered after CIA onset. This indicates that IL-23 is not critical during this stage of CIA. Interestingly and in line with our mouse data is a recent phase IIa, randomized, double-blind, placebo-controlled trial of an IL-12/IL-23 inhibitor in patients with rheumatoid arthritis that showed no clinical improvement in RA [11]. However, it has been shown that IL-17A is still involved in the pathophysiology of CIA at this stage of the disease and IL-17 blocking in RA looks promising [12]–[14]. This may indicate uncoupling of the IL-23/IL-17A axis during this stage of CIA and further studies will be needed to examine this hypothesis.

Murphy et al. have shown that IL-23 is critical in the formation of CD4+IL-17+ T cells [8]. In the present study, we showed that IL-23 is redundant for the maintenance of CII-specific IL-17A production in autoimmune arthritis. We found the highest proportion of splenic IL-17A-expressing CD4+ T cells 10 days after CII/CFA-immunization, showing that immunization with type II collagen (CII) induced the generation of Th17 cells. However, IL-23 is not required for CII-primed IL-17-producing CD4+ T cells to produce IL-17 after Ag-specific re-stimulation since IL-23 levels could not be measured and neutralization of IL-23 had no effect on IL-17 production (data not shown). In addition, adding IL-23 after the formation of CII-specific IL-17A-producing cells did not specifically enhance IL-17A in contrast to IFN-γ levels.

In vivo neutralizing IL-23 after CII immunization but before the onset of CIA significantly suppressed the severity of the disease. This was examined during the stage of CIA where CII-specific CD4+ IL-17+ T cells were already formed which is quite different compared to the IL-23p19 deficient mice, since these knock-out mice lack IL-17A-expressing CD4+ T cells from the start [8]. Neutralizing IL-23p19 after the induction of CII-specific of IL-17A-expressing CD4+ T cells did not prevent the onset of CIA as was described for the IL-23p19 deficient mice [8], and no difference was noted in disease incidence between anti-IL-23p19 treated mice and the control group. However, mice that developed CIA showed a significantly lower severity than control mice, indicating that IL-23 is involved in the regulation of disease progression during this stage of CIA. Another plausible explanation for the reduced disease severity in mice treated with anti-IL-23p19 before CIA onset is the potential role of IL-23 in the generation of pathogenic Th17 cells [15]–[17]. IL-23 suppresses IL-10 production by IL-6/TGFβ-differentiated Th17 cells [18] as well as from ex-vivo isolated CII-primed CD4+ T cells (Lubberts et al., unpublished observations). Additionally, exogenous IL-23 enhances the production of IL-17A and IL-22 by CD4+ CII-primed T cells by down-regulating T-bet and FoxP3 and up-regulating RORγt [19]. Altogether, this strongly suggests that IL-23 drives full maturation of pathogenic Th17 cells in vivo. Whether the Th17 cells have less pathogenic behavior after neutralizing IL-23p19 is at present under investigation.

The levels of CII-specific IgG’s were influenced after neutralizing IL-23 during onset of CIA. Both IgG1 and IgG2a/2b were lowered indicating no specific regulation of B cell activity. From this study it is not clear whether IL-23 had a direct effect on B cell activity and CII-specific IgG formation or indirectly. No difference in expression of B cell activation factors such as BAFF and APRIL were noted (data not shown). In contrast, no difference in the levels of CII-specific IgG’s was noted after blocking IL-23 after onset of CIA which was accompanied with no clinical improvement of the disease during this stage of CIA. Thus, neutralizing IL-23 before disease onset effectively suppressed disease severity whereas anti-IL-23p19 treatment of mice after CIA onset did not ameliorate disease. This strongly suggests IL-23-dependent and -independent stages of CIA. In experimental autoimmune encephalomyelitis (EAE; a mouse model for human multiple sclerosis), however, anti-IL-23p19 effectively ameliorated EAE when treatment was started at disease onset, peak of disease, or even during the first remission (Kikly et al., unpublished data). This difference in therapeutic efficacy of anti-IL-23p19 may be explained by the fact that the later stages of EAE autoimmunity are highly T cell dependent as is less so in CIA. Indeed, our current data suggests that neutralizing IL-23 mainly dampens T cell activity and the lower anti-CII antibodies in mice treated with anti-IL-23p19 before, but not after onset of CIA highlighting the role of IL-23 on T cell dependent B cell activation [20].

To further dissect the role of IL-23 on memory T cell dependent pathology, we used an antigen-induced arthritic (AIA) flare-up model in which memory T cells are responsible for joint inflammation [21]. Previously, we have shown that the primary antigen-induced arthritis (AIA) model is IL-23/IL-17 mediated, since IL-23p19- as well as IL-17RA-deficient mice were protected from progressive joint inflammation [22], [23]. Here, we started treatment of anti-IL-23p19 antibody after the primary AIA was declined and no clinically joint inflammation was observed. Persistent blocking of IL-23 activity before inducing the AIA flare-up significantly prevented the expression of severe joint inflammation with lower synovial mRNA expression of IL-17A and IL-22. This shows that memory T cell driven flare-up arthritis is IL-23-dependent, a phenomenon comparable to the prevention of EAE relapses with anti-IL-23p19 or anti-IL-23R [16], [24].

In summary, we have shown IL-23-dependent and IL-23-independent stages during autoimmune CIA. IL-23 is not a critical factor during the effector stage of CIA. In contrast, the memory T cell mediated flare-up arthritis is IL-23-mediated. These data suggest that specific neutralization of IL-23 using an anti-IL-23p19 antibody after onset of autoimmune arthritis may not be beneficial as a therapeutic therapy for RA patients. However, T cell mediated arthritis relapses in patients with RA might be controlled by anti-IL-23p19 treatment.

## Materials and Methods

### Ethics Statement

This study was carried out in strict accordance with the recommendations of the Dutch Animal ethics committee. The in vivo study protocol was approved by the Committee on the Ethics of Animal Experiments of the Erasmus MC (DEC nr. 128-10-03).

### Collagen-induced Arthritis

Male DBA/1 mice (Harlan, Horst, the Netherlands) were immunized intradermally with (100 µL of 1 mg/mL) bovine collagen type 2 (CII) (Chondrex) emulsified in complete Freund’s adjuvant (CFA). On day 21, mice were given an intra-peritoneal (i.p.) injection of 100 µg CII in PBS. CIA severity was assessed as previously described [12].

Mice were given 100 µg neutralizing murine IgG1 anti-murine IL-23, p19 specific (anti-IL-23p19) or murine IgG1 isotype control antibody i.p. on days 15 (before disease onset), 22 and 29. For anti-IL-23p19 antibody, the IC50 is 60 ng/ml as determined in a mouse splenocyte assay stimulated with mouse IL-23 and as a read out IL-17A was measured by ELISA in the culture supernatant. The BIAcore affinity determination at 37°C degrees gave a KD of 100 pM. The 3 mg/kg/week dose (i.p.) gave the best neutralization of disease progression in EAE (personal communications with Dr. K. Kikly). Alternatively, mice were observed daily and as soon as an arthritis score of 0.5–1.5 was observed, 100 µg anti-IL-23p19 or isotype control antibody was injected i.p. weekly for 3 or 6 weeks. All experiments were approved by the Dutch Animal ethics committee (DEC), protocol 128-10-03.

### Assessment of Collagen-induced Arthritis

Mice were considered to have arthritis when significant changes in redness and/or swelling were noted in the digits or in other parts of the paws. Arthritis was scored visually using the following scale: 0, noninflamed; 1, mild inflammation; 1.5, marked inflammation; 2, severe inflammation. Scoring was done by two independent observers, without knowledge of the experimental groups (9).

### Antigen-induced Arthritis

To induce antigen-induced arthritis (AIA), methylated BSA (mBSA, 8 mg/mL) was emulsified in an equal volume of CFA containing 1 mg/mL heat-killed M. tuberculosis (H37Ra; Difco). C57BL/6 Mice were immunized intradermally with 100 µL mBSA/CFA. One week later, 30 µg mBSA was injected intra-articular to induce mono-arthritis. Four weeks after the induction of the mono-arthritis, 100 µg anti-IL-23p19 or control antibody was injected ip, weekly for 5 weeks. Finally, one day after the last injection of anti-IL-23p19, a local arthritic flare was induced by injecting 2 µg mBSA intra-articular. One day later, mice were sacrificed and the severity of arthritis in the knee joint was scored macroscopically on a scale of 0–2 after removing the skin (0, no inflammation; 1, mild inflammation; 2, severe inflammation).

### Anti-collagen Antibody ELISAs

To determine anti-CII-specific antibodies, wells of microtiter plates were coated with 1 µg/mL CII, washed and blocked with 10% FCS. Samples and a reference-sample were serially diluted. Wells were washed, incubated with goat-anti-mouse IgG1, IgG2a or IgG2b-biotinylated antibodies (0.5 µg/mL) (Southern Biotechnology Associated, Inc.) and optical density at 450 nm was measured.

Levels of IL-17A (R&D Systems, Minneapolis, MN, USA) and IFN-γ (BD OptEIA™, BD Biosciences, Sunnyvale, CA, USA) were measured by ELISA according to the manufacturers’ instructions.

### Isolation of Ankle Infiltrating Cells

Ankles were cut into smaller pieces and put into 5 mL serum-free medium containing 0.65 Wünsch units Liberase TM Research Grade (Roche Applied Science, Indianapolis, In, USA). After 2 hours, cells were pushed through a 100 µm filter and these ankle-cells were processed for further use.

### Flow Cytrometric Analyses

For intracellular detection of cytokines, cells were stimulated with PMA (0.05 µg/mL; Sigma-Aldrich) and Ionomycin (0.5 µg/mL; Invitrogen) in the presence of GolgiStop™ (BD Biosciences, Sunnyvale, CA, USA) for 4 h. Cells were fixed using 2% PFA and permeabilized with 0.5% saponin (Sigma-Aldrich). Cells were stained with CD4-PerCP-Cy5.5 (RM4-5), IL-17A-Pe (TC11-18H10) and IFN-γ-Pe-Cy7 (XMG1.2) (all BD Pharmingen). Samples were acquired on a BD FACS CANTO II flow cytometer and analyzed using FlowJo (Tree Star, Inc.) software.

### Quantitative PCR

Total RNA of splenocytes was extracted, and DNaseI-treated RNA was used for cDNA synthesis (8). PCR primers were designed manually or using ProbeFinder software (Roche Applied Science, Indianapolis, In, USA) and probes were chosen from the Universal probe library (Roche Applied Science) (Table 1). Quantitative realtime PCR was performed using the ABI Prism 7900HT sequence-detection system (Applied Biosystems, Foster City, CA, USA) and analyzed using SDS v2.3 software (Applied Biosystems). The Ct values obtained were normalized to those of glycereraldehyde-3-phosphate dehydrogenase (GAPDH).

**Table 1:** Primers used in this study.

Gene	Sequence (5′ to 3′)	Probe nr*
Gapdh	F: AGCTTGTCATCAACGGGAAGR: TTTGATGTTAGTGGGGTCTCG	9
Il17a	F: TTTCCTGACCAAACTCAGCAR: TTCATTGTGGAGGGCAGAC	34
Il22	F: TTTCCTGACCAAACTCAGCAR: CTGGATGTTCTGGTCGTCAC	17

* refers to the Roche Universal probe library kit.

### In vitro Restimulation Assay

Splenocytes 10 days after bovine CII-immunization DBA/1 mice were isolated. MACS-isolated (negative selection using CD11b, GR-1, CD8, Ter119, TCRγδ, B220 and NK1.1 biotinylated antibodies) CD4+ T cells at a 4∶1 ratio with 50 Gy irradiated APCs were cultured for 3 days in the presence or absence of 50 µg/mL bovine CII with or without 50 ng/mL IL-23 (R&D Systems). After culture, cytokines were measured in supernatant by ELISA.

### Statistical Analyses

Differences between groups were tested with the Mann-Whitney U test. P values less than 0.05 were considered significant. Error bars are presented as standard error of the mean (SEM).

## Acknowledgments

We would like to thank N. Davelaar, M. Brem (Department of Rheumatology, Erasmus MC, Rotterdam), and N. Kops (Department of Orthopedics, Erasmus MC, Rotterdam), and Yara Hofman (Animal Care Facility, Erasmus MC, Rotterdam) for excellent technical assistance at various stages of the project.

## References

[1] Oppmann B, Lesley R, Blom B, Timans JC, Xu Y, et al (2000) Novel p19 protein engages IL-12p40 to form a cytokine, IL-23, with biological activities similar as well as distinct from IL-12. Immunity 13: 715–725. 11114383 10.1016/s1074-7613(00)00070-4

[2] Croxford AL, Mair F, Becher B (2012) IL-23: one cytokine in control of autoimmunity. Eur J Immunol 42: 2263–2273. 22949325 10.1002/eji.201242598

[3] Parham C, Chirica M, Timans J, Vaisberg E, Travis M, et al (2002) A receptor for the heterodimeric cytokine IL-23 is composed of IL-12Rbeta1 and a novel cytokine receptor subunit, IL-23R. J Immunol 168: 5699–5708. 12023369 10.4049/jimmunol.168.11.5699

[4] Mangan PR, Harrington LE, O’Quinn DB, Helms WS, Bullard DC, et al. (2006) Transforming growth factor-beta induces development of the T(H)17 lineage. Nature. 10.1038/nature04754 16648837

[5] Kolls JK, Linden A (2004) Interleukin-17 family members and inflammation. Immunity 21: 467–476. 15485625 10.1016/j.immuni.2004.08.018

[6] Hunter CA (2005) New IL-12-family members: IL-23 and IL-27, cytokines with divergent functions. Nat Rev Immunol 5: 521–531. 15999093 10.1038/nri1648

[7] Wiekowski MT, Leach MW, Evans EW, Sullivan L, Chen SC, et al (2001) Ubiquitous transgenic expression of the IL-23 subunit p19 induces multiorgan inflammation, runting, infertility, and premature death. J Immunol 166: 7563–7570. 11390512 10.4049/jimmunol.166.12.7563

[8] Murphy CA, Langrish CL, Chen Y, Blumenschein W, McClanahan T, et al (2003) Divergent pro- and antiinflammatory roles for IL-23 and IL-12 in joint autoimmune inflammation. J Exp Med 198: 1951–1957. 14662908 10.1084/jem.20030896 PMC2194162

[9] Yago T, Nanke Y, Kawamoto M, Furuya T, Kobashigawa T, et al (2007) IL-23 induces human osteoclastogenesis via IL-17 in vitro, and anti-IL-23 antibody attenuates collagen-induced arthritis in rats. Arthritis Res Ther 9: R96. 17888176 10.1186/ar2297 PMC2212562

[10] Yanaba K, Hamaguchi Y, Venturi GM, Steeber DA, St Clair EW, et al (2007) B cell depletion delays collagen-induced arthritis in mice: arthritis induction requires synergy between humoral and cell-mediated immunity. J Immunol 179: 1369–1380. 17617630 10.4049/jimmunol.179.2.1369

[11] Krausz S, Boumans MJ, Gerlag DM, Lufkin J, van Kuijk AW, et al (2012) Brief report: a phase IIa, randomized, double-blind, placebo-controlled trial of apilimod mesylate, an interleukin-12/interleukin-23 inhibitor, in patients with rheumatoid arthritis. Arthritis Rheum 64: 1750–1755. 22170479 10.1002/art.34339

[12] Lubberts E, Koenders MI, Oppers-Walgreen B, van den Bersselaar L, Coenen-de Roo CJ, et al (2004) Treatment with a neutralizing anti-murine interleukin-17 antibody after the onset of collagen-induced arthritis reduces joint inflammation, cartilage destruction, and bone erosion. Arthritis Rheum 50: 650–659. 14872510 10.1002/art.20001

[13] Genovese MC, Van den Bosch F, Roberson SA, Bojin S, Biagini IM, et al (2010) LY2439821, a humanized anti-interleukin-17 monoclonal antibody, in the treatment of patients with rheumatoid arthritis: A phase I randomized, double-blind, placebo-controlled, proof-of-concept study. Arthritis Rheum 62: 929–939. 20131262 10.1002/art.27334

[14] Hueber W, Patel DD, Dryja T, Wright AM, Koroleva I, et al (2010) Effects of AIN457, a fully human antibody to interleukin-17A, on psoriasis, rheumatoid arthritis, and uveitis. Sci Transl Med 2: 52ra72. 10.1126/scitranslmed.3001107 20926833

[15] Gyulveszi G, Haak S, Becher B (2009) IL-23-driven encephalo-tropism and Th17 polarization during CNS-inflammation in vivo. Eur J Immunol 39: 1864–1869. 19544494 10.1002/eji.200939305

[16] McGeachy MJ, Chen Y, Tato CM, Laurence A, Joyce-Shaikh B, et al (2009) The interleukin 23 receptor is essential for the terminal differentiation of interleukin 17-producing effector T helper cells in vivo. Nat Immunol 10: 314–324. 19182808 10.1038/ni.1698 PMC2945605

[17] Thakker P, Leach MW, Kuang W, Benoit SE, Leonard JP, et al (2007) IL-23 is critical in the induction but not in the effector phase of experimental autoimmune encephalomyelitis. J Immunol 178: 2589–2598. 17277169 10.4049/jimmunol.178.4.2589

[18] McGeachy MJ, Bak-Jensen KS, Chen Y, Tato CM, Blumenschein W, et al (2007) TGF-beta and IL-6 drive the production of IL-17 and IL-10 by T cells and restrain T(H)-17 cell-mediated pathology. Nat Immunol 8: 1390–1397. 17994024 10.1038/ni1539

[19] Mus AM, Cornelissen F, Asmawidjaja PS, van Hamburg JP, Boon L, et al (2010) Interleukin-23 promotes Th17 differentiation by inhibiting T-bet and FoxP3 and is required for elevation of interleukin-22, but not interleukin-21, in autoimmune experimental arthritis. Arthritis Rheum 62: 1043–1050. 20131264 10.1002/art.27336

[20] Ghilardi N, Kljavin N, Chen Q, Lucas S, Gurney AL, et al (2004) Compromised humoral and delayed-type hypersensitivity responses in IL-23-deficient mice. J Immunol 172: 2827–2833. 14978083 10.4049/jimmunol.172.5.2827

[21] Lens JW, van den Berg WB, van de Putte LB, Berden JH, Lems SP (1984) Flare-up of antigen-induced arthritis in mice after challenge with intravenous antigen: effects of pre-treatment with cobra venom factor and anti-lymphocyte serum. Clin Exp Immunol 57: 520–528. 6235993 PMC1536280

[22] Cornelissen F, Mus AM, Asmawidjaja PS, van Hamburg JP, Tocker J, et al (2009) Interleukin-23 is critical for full-blown expression of a non-autoimmune destructive arthritis and regulates interleukin-17A and RORgammat in gammadelta T cells. Arthritis Res Ther 11: R194. 20017902 10.1186/ar2893 PMC3003524

[23] Lemos HP, Grespan R, Vieira SM, Cunha TM, Verri WA Jr, et al (2009) Prostaglandin mediates IL-23/IL-17-induced neutrophil migration in inflammation by inhibiting IL-12 and IFNgamma production. Proc Natl Acad Sci U S A 106: 5954–5959. 19289819 10.1073/pnas.0812782106 PMC2667068

[24] Chen Y, Langrish CL, McKenzie B, Joyce-Shaikh B, Stumhofer JS, et al (2006) Anti-IL-23 therapy inhibits multiple inflammatory pathways and ameliorates autoimmune encephalomyelitis. J Clin Invest 116: 1317–1326. 16670771 10.1172/JCI25308 PMC1450386

